# Hepatitis B Surface Antigen Quantity Positively Correlates with Plasma Levels of microRNAs Differentially Expressed in Immunological Phases of Chronic Hepatitis B in Children

**DOI:** 10.1371/journal.pone.0080384

**Published:** 2013-11-11

**Authors:** Thilde Nordmann Winther, Ida Louise Heiberg, Claus Heiner Bang-Berthelsen, Flemming Pociot, Birthe Hogh

**Affiliations:** 1 Department of Paediatrics, Hvidovre Hospital, University of Copenhagen, Copenhagen, Denmark; 2 Diagnostic Unit and Center for Non-Coding RNA in Technology and Health, Glostrup Research Institute, Glostrup Hospital, University of Copenhagen, Glostrup, Denmark; University of Cincinnati College of Medicine, United States of America

## Abstract

**Background and Aim:**

Children with chronic hepatitis B (CHB) are at high risk of progressive liver disease. It is suggested that a newly-identified panel of 16 microRNAs is important in the pathogenesis of CHB in children. Subviral hepatitis B surface antigen (HBsAg) particles are produced in large excess over infectious virions. Interestingly, circulating HBsAg particles have been shown to carry microRNAs. A thorough characterisation of the identified microRNAs and HBsAg over time in plasma from children with CHB may provide useful information about the natural course of childhood CHB.

**Patients and Methods:**

A cohort of 42 children with CHB was followed over time. Three to five blood samples were obtained from each child at minimum intervals of half a year; in total 180 blood samples. Plasma levels of the 16 microRNAs previously identified were analysed by quantitative real-time polymerase-chain-reaction. Plasma HBsAg was quantified using ARCHITECT® HBsAg assay.

**Results:**

The presence of 14/16 plasma microRNAs in children with CHB was confirmed. All 14 microRNAs were significantly differentially expressed in different immunological phases of the disease. MicroRNA plasma levels were highest in immune-tolerant children, lower in immune-active children, and reached the lowest values in immune-inactive children, p<0.001. Plasma levels of four microRNAs decreased significantly over time in immune-tolerant and immune-active children whereas the microRNA plasma levels were stable in immune-inactive children, p<0.004. HBsAg quantity was positively correlated with plasma levels of 11/14 microRNAs, p<0.004.

**Conclusion:**

This is the first study to characterise plasma microRNAs and HBsAg over time in children with CHB. Our data suggest that plasma levels of selected microRNAs and HBsAg are inversely correlated with immunological control of CHB in children. Further studies are, however, needed to advance the understanding of microRNAs and HBsAg in the pathogenesis of CHB in children.

## Introduction

Children with chronic hepatitis B (CHB) have a lifetime risk of developing hepatocellular carcinoma up to 25%, and an incidence of cirrhosis of 2-3% per year [1]. Life-long follow-up is warranted, and treatment is recommended if signs of liver damage are detected [2]. Despite recent advances and developments in anti-viral therapies no treatment available is consistently effective in curing CHB in children [3]. Improved knowledge about the immunopathogenesis of CHB is essential to advance management of children with CHB.

The natural course of CHB is dynamic and depends on the complex interplay between virus and host. Typically, chronic infection acquired during infancy has three phases: the immune-tolerant, the immune-active, and the immune-inactive phase. The immunological phases are clinically characterised by the persistence of hepatitis B e antigen (HBeAg) together with levels of hepatitis B virus (HBV) DNA and alanine aminotransferases (ALT). Children in the inactive phase have a low risk of liver disease progression, but HBV reactivation can occur and trigger immune-mediated liver injury (HBeAg-negative CHB) [4]. The mechanisms regulating the immune response are yet to be fully understood.

MicroRNAs affect the outcome of the immune response to infections [5]. Accumulated evidence suggests that the pathogenesis of HBV infection is also modulated by microRNAs [6]. Our group recently investigated the plasma microRNA profile of children with CHB and of healthy controls in a cross-sectional study. We identified a panel of 16 plasma microRNAs aberrantly expressed in children with CHB. Levels of all the microRNAs were significantly higher in plasma from HBeAg-positive children when compared to HBeAg-negative children [7]. The majority of the microRNAs have previously been shown to associate with CHB in adults [8-10].

This newly identified panel of microRNAs may be of particular importance in the pathogenesis of CHB in children. However, a more thorough characterisation of the microRNAs in the natural course of the disease is essential to advance understanding of the role played by these microRNAs.

It has been suggested that hepatitis B surface antigen (HBsAg) is involved in the orchestration of the immune response [11,12]. HBsAg in plasma is derived from virions and subviral HBsAg particles. Subviral particles are secreted from infected hepatocytes in quantities that outnumber virions by several orders of magnitude [13]. The biological function of this apparent excess-production is uncertain. To our knowledge, no studies have investigated plasma HBsAg quantity in the natural course of CHB in children.

Recent studies indicate that microRNAs interact with HBsAg. For example, subviral HBsAg particles were shown to carry hepatocellular microRNAs [14], and a study in adults with CHB revealed a significant correlation between levels of circulating HBsAg and miR-122 [15]. This interesting relationship warrants further investigation.

The present study aimed to characterise the 16 microRNAs identified in our previous study and their correlation to HBsAg in the natural course of childhood CHB. Presence of 14 out of 16 plasma microRNAs in children with CHB was confirmed. All 14 microRNAs were significantly differentially expressed in different immunological phases of the disease. MicroRNA plasma levels were highest in immune-tolerant children, lower in immune-active children, and reached the lowest values in immune-inactive children. Plasma levels of four microRNAs decreased significantly over time in immune-tolerant and immune-active children whereas the microRNA plasma levels were stable in immune-inactive children. HBsAg quantity correlated positively with microRNA plasma levels.

## Patients and Methods

### Patients

The definition of CHB is HBsAg seropositivity for more than six months [4] and CHB is a notifiable disease in Denmark [16]. Danish children with CHB are mainly adoptees and immigrants from high endemic countries.

Since May 2000 all children aged 0-16 years who reported to Statens Serum Institut, Copenhagen, Denmark with CHB have been invited by letter to participate in a cohort of Danish children with CHB. Inclusion is consecutively and ongoing. Up until the inclusion deadline of this study (February 2011) a total of 202 children with CHB were invited to participate in the cohort and a total of 60 children were included in the cohort. The children are followed in accordance with international guidelines [2,4,17]. Blood samples are obtained at regular clinical visits every third, sixth, or twelfth month. Medical records are obtained and examined for all of the children.

In the present study, participating children were selected from the cohort of Danish children with CHB. Inclusion criteria: The child 1) were included in the cohort of Danish children with CHB during the period of May 2006 to February 2011, and 2) provided at least three blood samples with minimum intervals of half a year from inclusion up until August 2012. No exclusion criteria. A total of 42 children with CHB met the criteria and were included in the present study.

Of the 42 children included, 23 were followed at the Department of Paediatrics, Hvidovre Hospital, University of Copenhagen, Denmark and 19 were followed at outpatient clinics at hospitals elsewhere in Denmark. 

### Ethical considerations

The study was performed in accordance with the criteria of the Helsinki II Declaration and was approved by the Ethics Committee, Capital Region of Denmark, Reference Number H-KF-255584 and the Danish Data Protection Agency, Journal Number 2009-41-4193. Parents of all participants provided informed written consent prior to any study procedure.

### Blood samples

Blood samples were collected in EDTA tubes. In Denmark it is standard procedure to process blood samples for plasma isolation within four hours of collection. All blood samples collected at Hvidovre Hospital, University of Copenhagen, Denmark were processed according to standard procedure (samples from 23/42 children with CHB). The remaining 19/42 children with CHB were followed at outpatient clinics elsewhere in Denmark. For logistic reasons, it was not possible to process the blood samples for plasma isolation at all of the outpatient clinics. Therefore, to ensure standardised handling all blood samples were sent by mail (at ambient temperature) to the Department of Clinical Biochemistry, Hvidovre Hospital, University of Copenhagen, Denmark for further processing. This implied a processing time of up to 48 hours from collection of blood samples to plasma isolation. Upon arrival at the Department of Clinical Biochemistry, Hvidovre Hospital, University of Copenhagen, Denmark all blood samples were centrifuged at 2500 g for 10 minutes, separated, aliquoted, and stored at -80°C [7].

### Virology and clinical chemistry

#### Serological status

Data on serological status (HBeAg, anti-HBeAg, anti-HAV, anti-HCV, and anti-HIV) were obtained from the medical records of the children.

#### HBsAg quantification

HBsAg was quantified using ARCHITECT® HBsAg assay (a two-step chemiluminescent microparticle immunoassay) (Abbott, Chicago, IL, USA) according to manufacturer’s instructions. In the first step, sample and anti-HBs coated paramagnetic microparticles were combined. HBsAg in the sample was bound to the anti-HBs coated microparticles. After washing, Acridium-labeled anti-HBs conjugate was added in the second step. Following another wash cycle, Pre-Trigger and Trigger Solutions were added to the reaction mixture. The resulting chemiluminescent reaction was measured as relative light units. A direct relationship exists between the amount of HBsAg in the sample and the relative light units detected by the ARCHITECT i* System optics. The concentration of HBsAg was determined using an internal ARCHITECT HBsAg calibration curve. The lower limit of detection was 0.05 IU/ml [18,19]. HBsAg quantification was performed at the Department of Clinical Biochemistry, Aalborg Hospital, Aarhus University, Denmark.

#### HBV DNA quantification

The levels of HBV DNA were measured using quantitative real-time polymerase chain reaction (qRT-PCR). In brief, total nucleic acids were extracted from 200 µl plasma on a MagnaPure 96 extraction robot, using the MagNA Pure 96 DNA and Viral NA small volume kit and the plasma small volume protocol choosing, elution volume 100 µl (Roche, Basel, Switzerland). qRT-PCR was performed on a Stratagene Mx3005 qPCR system (Agilent Technologies, Santa Clara, California, USA) using 15 µl of extraction in a total reaction volume of 50 µl. The assay contained QuantiTect Probe PCR kit (Qiagen, Hilden, Germany) mixed with 600 nM forward primer HBCPF (5´-ATCTTATCAACACTTCCGGA-3´, nucleotides 2315-2334), 800nM reverse primer HBCPR (5´-AGATTGAGATCTTCTGCGAC-3´, nucleotides 2434-2415), and 150nM FAM labelled Taqman Probe HBCPP (5´-FAM-AGGTCCCCTAGAAGAAGAACTCCCT-TAMRA-3´, nucleotides 2360-2384), targeting a 120 bp region of the core protein gene in the hepatitis B virus. Cycling conditions were: 2 minutes at 50°C and 15 minutes at 95°C; 40 cycles of 15 seconds at 95°C and 1 minute at 60°C. The quantification was based on an absolute standard curve made from a HepG2-2.2.15 cell-suspension, which was calibrated using the WHO standard NIBSC (97/750). Over 90% of the assays detect a minimum of HBV DNA 50 IU/ml whereas 20% of the assays detect HBV DNA 25 IU/ml [20]. HBV DNA quantification was conducted at Statens Serum Institut, Copenhagen, Denmark.

#### HBV genotyping

HBV genotyping was performed by PCR as previously described [21]. Genotype-specific primer pairs were selected from the pre-S region of the HBV genome, amplifying PCR products of approximately 250 base pairs. The lower detection limit of the assay is HBV DNA <30 IU/ml. Genotyping was performed at the Department of Clinical Biochemistry, Aalborg Hospital, Aarhus University, Denmark. 

#### ALT

Levels of ALT were obtained from the medical records of the children.

### MicroRNA analyses

#### RNA extraction

From each plasma sample, 250 µL of plasma was centrifuged at 3000 g for 5 minutes, and the upper 200 μL of plasma was used for RNA extraction. The total RNA was extracted from plasma using the Qiagen miRNeasy® Mini Kit (Qiagen, Hilden, Germany) in accordance with the manufacturer’s instructions with minor modifications: 1.25 μg/mL of MS2 bacteriophage RNA (Roche, Basel, Switzerland) was added to the QIAzol Lysis Reagent, and washing with RPE buffer was repeated x3 instead of x2. RNA extractions were performed in duplicate. Exiqon Services, Vedbaek, Denmark conducted the extractions. RNA was stored at -80°C until analysis. 

#### qRT-PCR

2 µL total RNA was reverse transcribed in 10µL reactions using the miRCURY LNA^TM^ Universal RT microRNA PCR, Polyadenylation and cDNA synthesis kit (Exiqon, Vedbaek, Denmark) according to the manufacturer’s instructions. cDNA was diluted x50 and assayed in 10 µL PCR reactions according to the protocol for miRCURY LNA™ Universal RT microRNA PCR; each microRNA was assayed once by qRT-PCR on the Pick-&-Mix microRNA PCR panel (Exiqon, Vedbaek, Denmark). Negative controls excluding template from the reverse transcription reaction were performed and profiled like the samples. The amplification was performed in a LightCycler® 480 Real-Time PCR System (Roche, Basel, Switzerland) in 384 well plates. The amplification curves were analysed using the Roche LC software, both for determination of cycle threshold (C_T_) values and for melting curve analyses. Over 80% of the assays detect a minimum of 100 microRNA copies in the qRT-PCR reaction, whereas close to 50% detect 10 microRNA copies [22]. qRT-PCRs were performed at Exiqon Services, Vedbaek, Denmark.

#### Design of Pick-&-Mix microRNA PCR panels

The Pick-&-Mix microRNA PCR panels applied included pre-designed primers for 21 microRNAs and, in addition, Exiqon’s RNA spike-in UniSp6. Of the 21 microRNAs 16 were the microRNAs of interest: miR-99a-5p, -100-5p, -122-5p, -122-3p, -125b-5p, 192-5p, -192-3p, -193b-3p, -194-5p, -215, -365a-3p, -455-5p, -455-3p, -483-3p, -855-5p, and –1247-5p. These 16 microRNAs were previously identified as the most aberrantly-expressed in plasma from HBeAg-positive and HBeAg-negative children when compared to healthy controls in a cross-sectional study [7]. For normalisation of the data set, three microRNAs (miR-22-5p, -26a-5p, and –221-3p) were included as previously described [7,23,24]. To assess possible haemolysis in the samples, two microRNAs were used: miR-23a-3p and –451a. miR-23a-3p is relatively stable in plasma and not affected by haemolysis, whereas miR-451a is highly expressed in red blood cells. If the ΔC_T_ value of miR-451a - miR-23a-3p is lower than seven the sample is accepted but if it is higher there is a risk of haemolysis affecting the analyses [25,26]. UniSp6 was added in the reverse transcription reaction, providing an opportunity to evaluate the quality of the RT reaction. 

### Analysis of qRT-PCR data

The amplification efficiency was calculated using algorithms similar to the LinReg software. All assays were inspected for distinct melting curves, and the primer melting temperature was checked to be within known specifications for the assay. Data that did not pass these criteria were omitted from any further analysis. If not analysed the assays were excluded. The geometric mean of miR-22-5p, miR-26a-5p, and miR-221-3p was used for normalisation of the data set. The geometric mean C_T_-values from duplicates were calculated, and the comparative C_T_ method was used to analyse the data [27]. Results are shown as -ΔC_T_-values.

### Statistical analyses

Data were analysed using SAS software, version 9.2 (SAS Institute, Cary, NC, USA). Regression analyses were performed in two steps. First, a univariate model was used in which each parameter was added one at a time. Second, a multivariate model was created including all relevant parameters. Each microRNA was analysed separately. Repeated measurement analyses (proc mixed) on ranked data were used. Adjustment for multiplicity was performed, and p<0.004 was regarded as significant (Bonferroni).

## Results

### Patient characteristics

The study included 42 children with CHB: 16 boys (38%) and 26 girls (62%). Mean age at inclusion was 9.4 years (SD ±3.8 years, range 1.4 - 15.6 years). Twenty-nine were Asian (70%), 9 (21%) were Caucasian, and 4 (9%) were African. Twenty-seven children (64%) were infected with HBV vertically from mother-to-child. For the remaining 15 children (36%) the transmission route was unknown. None of the children showed any clinical signs of liver damage, and none had received antiviral treatment for their hepatitis. All patients were negative for HIV, hepatitis A, and hepatitis C virus. HBV genotypes were successfully determined in 36 of the 42 HBV-infected children (86%); one was genotype A (2%), 11 were genotype B (26%), five were genotype C (12%), 13 were genotype D (31%), five were genotype E (12%), and one was genotype F (2%). Genotyping was not feasible in six of 42 children (14%) due to HBV DNA levels below the detection limit (HBV DNA<30 IU/mL). These six children were all immune-inactive that is; only 54% of immune-inactive children were successfully genotyped. Genotypes were therefore excluded from further analyses.

The study was longitudinal, and mean follow-up time was 3.8 years (SD ±1.5 years, range 1.0 - 6.3 years). Three to five blood samples were obtained from each child at minimum intervals of half a year. In total, 180 blood samples were obtained. 

At inclusion, children with CHB were classified according to immunological phase of disease based on international guidelines [2,4,17]: 14 children were in the immune-tolerant phase, 14 were in the immune-active phase, and 13 were in the immune-inactive phase. Only one child with HBeAg-negative CHB showed signs of HBV reactivation (had elevated ALT (52-80 U/L) and low but fluctuating levels of HBV DNA (0-1E+03 IU/mL) and was excluded from any further analyses. Four children HBeAg-seroconverted during the study. They were initially classified in the immune-active phase but, approximately one year after seroconversion, classified in the immune-inactive phase. 

Accordingly, mean ALT and HBV DNA levels (±SD) in children classified in different immunological phases were as follows: immune-tolerant: ALT 27 ±8 U/L and HBV DNA 4E+08 ±1E+09 IU/mL; immune-active: ALT 66 ±45 U/L and HBV DNA 1E+08 ±2E+08 IU/mL; and immune-inactive: ALT 23 ±8 U/L and HBV DNA 1E+03 ±3E+03 IU/mL (one sample from a child in the immune-active phase showed ALT=1126 U/L, and the sample was excluded to avoid skewing the data set). 

Patient characteristics are summarised in Table 1.

**Table 1:** Patient characteristics.

	**Immunological phase of CHB**			
	**Tolerant**	**Active**	**Inactive**	**Active** → **Inactive**
**Number of children**	14	10	13	4
**Male/female**	5/9	2/8	5/8	3/1
**Race**	As=14	Af=2 As=5 C=3	Af=1 As=7 C=5	Af=1 As=2 C=1
**Mean age at inclusion ±SD**	8.5 ±3.2	9.2 ±4.6	11.7 ±2.5	5.2 ±2.9
**Mean follow-up years ±SD**	3.5 ±1.5	3.9 ±1.7	4.0 ±1.2	4.6 ±1.7
**Transmission**	Ver=10, Unk=4	Ver=8, Unk=2	Ver=4, Unk=9	Ver=4
**Genotypes**	B C D	A B C D E F	B C D E	C D E
**ALT I /U ±SD**	27 ±8	66 ±45	23 ±8	49 ±35
**HBV DNA ±SD**	4E+08 ±1E+09	1E+08 ±2E+08	1E+03 ±3E+03	2E+07 ±5E+07

Footnote: Asian (As), African (Af), Caucasian (C). Vertical (ver), Unknown (unk).

### MicroRNA analyses

qRT-PCR analyses of 16 selected target microRNAs were performed on 180 plasma samples. Controls (non-template control and RNA spike-in) indicated adequate technical performance of the experiments. To determine data quality crudely for each sample, the levels of microRNAs were compared in all samples. The samples were comparable in microRNA content, suggesting that the samples were of similar quality and were processed reproducibly. Analysis of haemolysis using the expression ratio between miR-451a and miR-23a-3p indicated that 6 of 180 samples analysed were affected by haemolysis. These samples were omitted from analyses. A small number of assays were excluded due to unacceptable data quality control (melting curve analysis and calculation of amplification efficiency). Likewise, if not analysed, assays were excluded. For miR-455-3p and miR-1247-5p, a large number of samples were not analysed and, therefore, these two microRNAs were excluded from any further analyses. In Table 2, the total number of samples successfully analysed for each of the selected target microRNAs is listed. 

**Table 2:** Total number of plasma samples successfully analysed by qRT-PCR for each of the selected 16 target microRNAs.

**MicroRNA**	**Samples successfully analysed**
99a-5p	173/180
100-5p	174/180
122-5p	174/180
122-3p	174/180
125b-5p	174/180
192-5p	174/180
192-3p	162/180
193b-3p	171/180
194-5p	174/180
215	174/180
365a-3p	174/180
455-5p	166/180
455-3p	119/180 *
483-3p	161/180
855-5p	173/180
1247-5p	104/180 *

Footnote: * Excluded from any further analyses.

Blood samples from 19/42 children with CHB were not processed according to standard procedure. The samples were shipped, which implied a delay in the processing time of up to 48 hours from collection to plasma isolation. To assess the impact of extended processing time on the plasma microRNA levels we compared the plasma levels of the 14 microRNAs in samples processed according to standard procedure (within four hours of collection) with in samples processed after a delay of up to 48 hours. No difference was found (Table S1) and the results of all the samples are presented together in the following.

### MicroRNA plasma levels in different immunological phases of CHB

**Figure 1:**
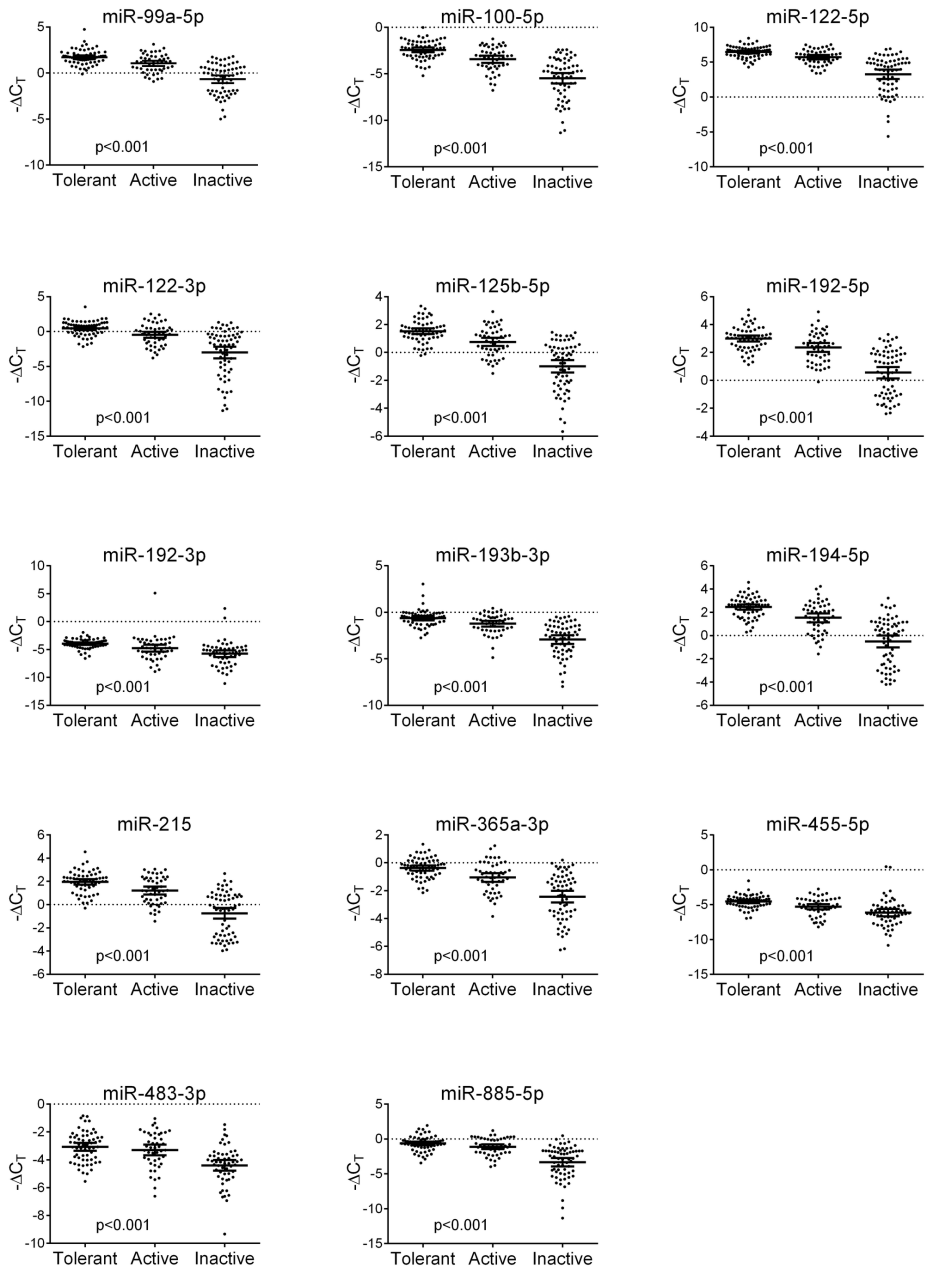
MicroRNA plasma levels in different immunological phases of CHB in children. Levels of 14 microRNAs were assessed in plasma from children with CHB. The children were classified according to immunological phase of disease based on international guidelines. All the microRNAs were consistently differentially expressed in different immunological phases of the disease, p<0.001. The correlations persisted when adjusted for age, gender, and race. Plasma levels of all 14 microRNAs were highest in immune-tolerant children, lower in immune-active children, and reached the lowest values in immune-inactive children. The range of plasma microRNA levels in immune-inactive children was wide and all the newly-seroconverted children showed plasma microRNA levels in the upper range. The bars represent geometric means -ΔC_T_ values ±SEM.

A total of 14 plasma microRNAs were included in the statistical analyses. We assessed the levels of these 14 microRNAs in plasma from children classified into different immunological phases of CHB. All the microRNAs were consistently differentially expressed between the three groups, p<0.001. The correlations persisted when corrected for age, gender, and race, p<0.001. Plasma levels of all 14 microRNAs were highest in immune-tolerant children, lower in immune-active children and lowest in immune-inactive children. The range of plasma microRNA levels in immune-inactive children was notably wide. Plasma microRNA levels in newly-seroconverted children were all in the upper range. Figure 1.

Due to the natural history of CHB the age of children classified info different immunological phases of the disease varies (Table 1). We therefore investigated the association between microRNA plasma levels and age. A significant negative correlation was identified for the majority of microRNAs, p<0.001 (did not apply for miR-192-3p, -455-5p, and –483-3p). Importantly, however, when adjusted for immunological phase no significant correlations were identified between microRNA plasma levels and age. We also investigated the relationships between microRNA plasma levels and gender and race. No associations were identified. 

The longitudinal study design enabled the characterisation of microRNA plasma levels over time. First, we analysed the developments within the individual child. Overall decreases in plasma levels of 11 out of 14 microRNAs were observed during the study period, p<0.001 (no significant decrease was observed for miR-192-3p (p=0.008), -455-5p (p=0.6), and -483-3p (p=0.9)). 

**Figure 2:**
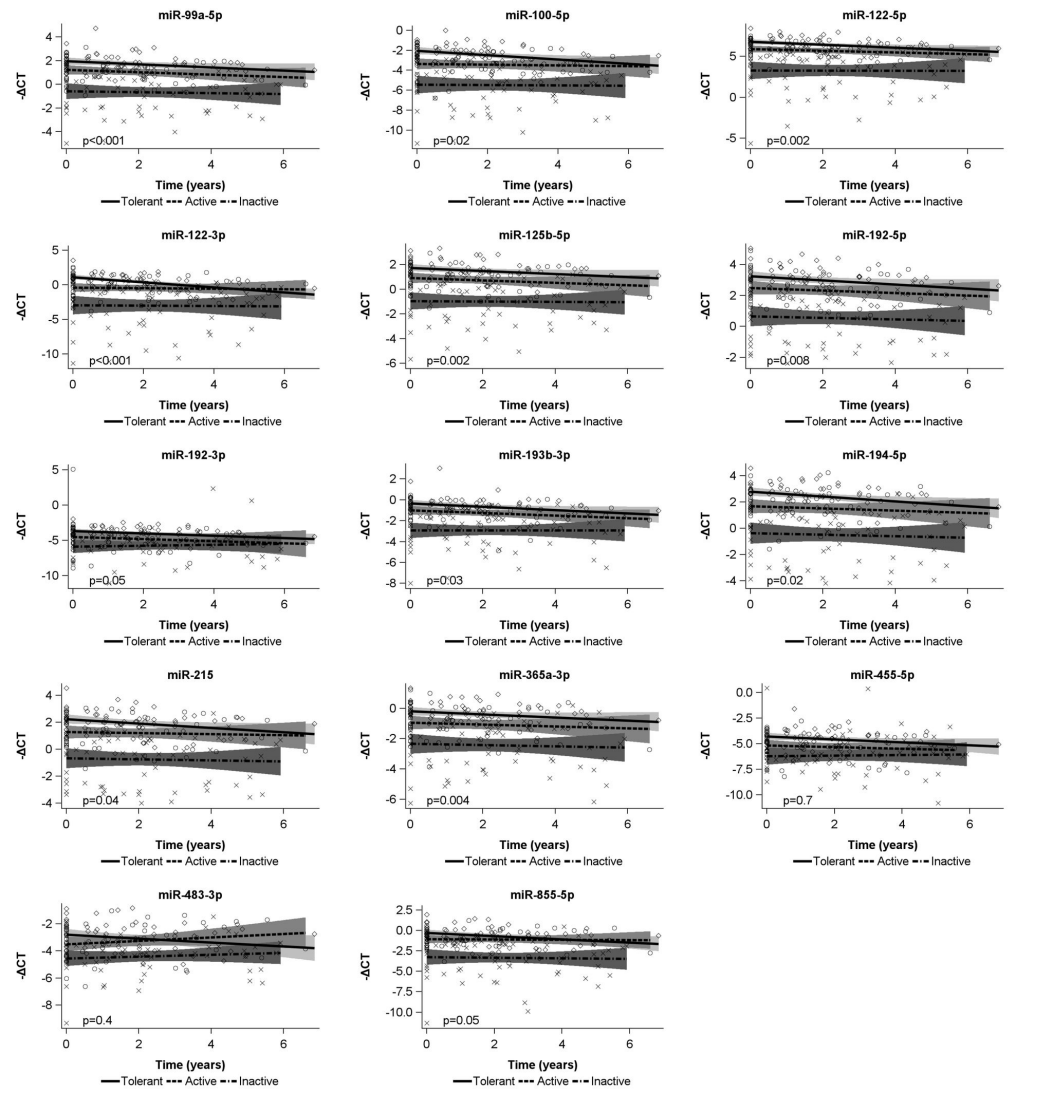
MicroRNA plasma levels over time in children classified into different immunological phases of CHB. We looked into the quantitative developments over time in children classified in different immunological phases of CHB. In plasma from immune-tolerant and immune-active children, decreases in microRNA plasma levels were identified, whereas microRNA plasma levels in immune-inactive children were stable. The correlations, however, were only significant for four of the microRNAs investigated: miR-99a-5p, -122-5p, -122-3p, and -125b-5p. Due to multiple testing only p<0.004 was regarded as significant. The results were adjusted for age. Bars: geometric means -ΔC_T_ values; marked area: 95% confidence interval; time (years): the period from collection of the first sample from a given child.

Next, we looked into the quantitative developments of plasma microRNAs over time in children classified in different immunological phases of CHB. In plasma from immune-tolerant and immune-active children, decreases in microRNA plasma levels were identified, whereas microRNA plasma levels in immune-inactive children were stable. The above correlations, however, were only significant for four of the microRNAs investigated: miR-99a-5p, -122-5p, -122-3p, and -125b-5p. The results were adjusted for age. Figure 2.

### MicroRNAs and ALT

**Figure 3:**
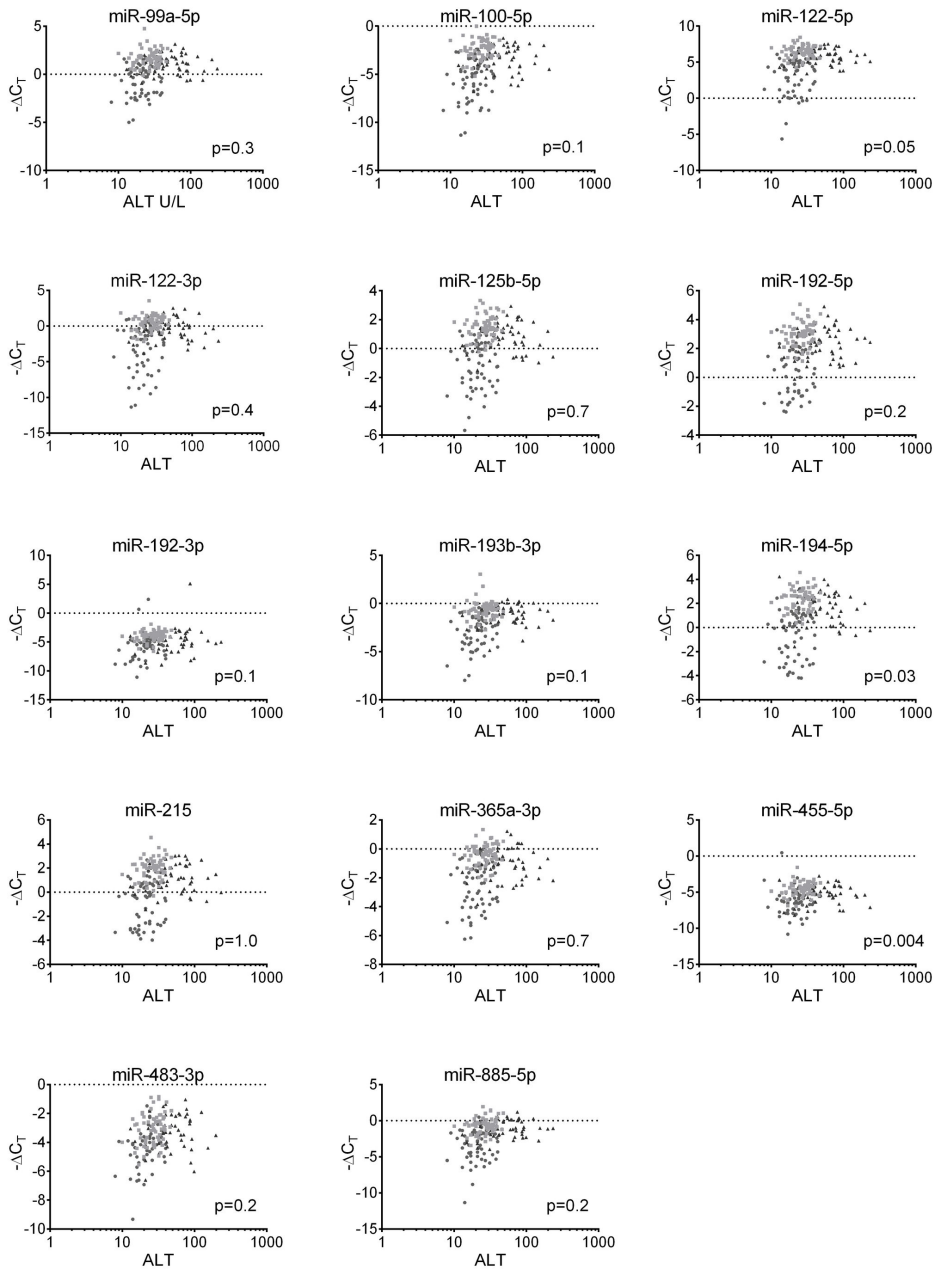
Correlations between plasma levels of microRNAs and ALT. The relationship between plasma levels of microRNAs and ALT was investigated. No significant correlations were identified. Light grey square: immune-tolerant; black triangle: immune-active; dark grey circle: immune-inactive. Due to multiple testing only p<0.004 was regarded as significant.

We looked into the association between plasma levels of microRNAs and ALT. No such correlations were observed for any of the microRNAs investigated. Figure 3. Interestingly, when analysing the correlation between plasma levels of microRNAs and ALT in immune-active and immune-inactive children only (excluding data on immune-tolerant children) two microRNAs, miR-122-5p and miR-885-5p, stood out showing low although not significant p-values; p=0.03 and p=0.04 for miR-122-5p and miR-885-5p, respectively. The remainder of microRNAs showed p-values between 0.2 and 1.0 (Due to multiple testing only p<0.004 was regarded as significant). 

### HBsAg quantity

**Figure 4:**
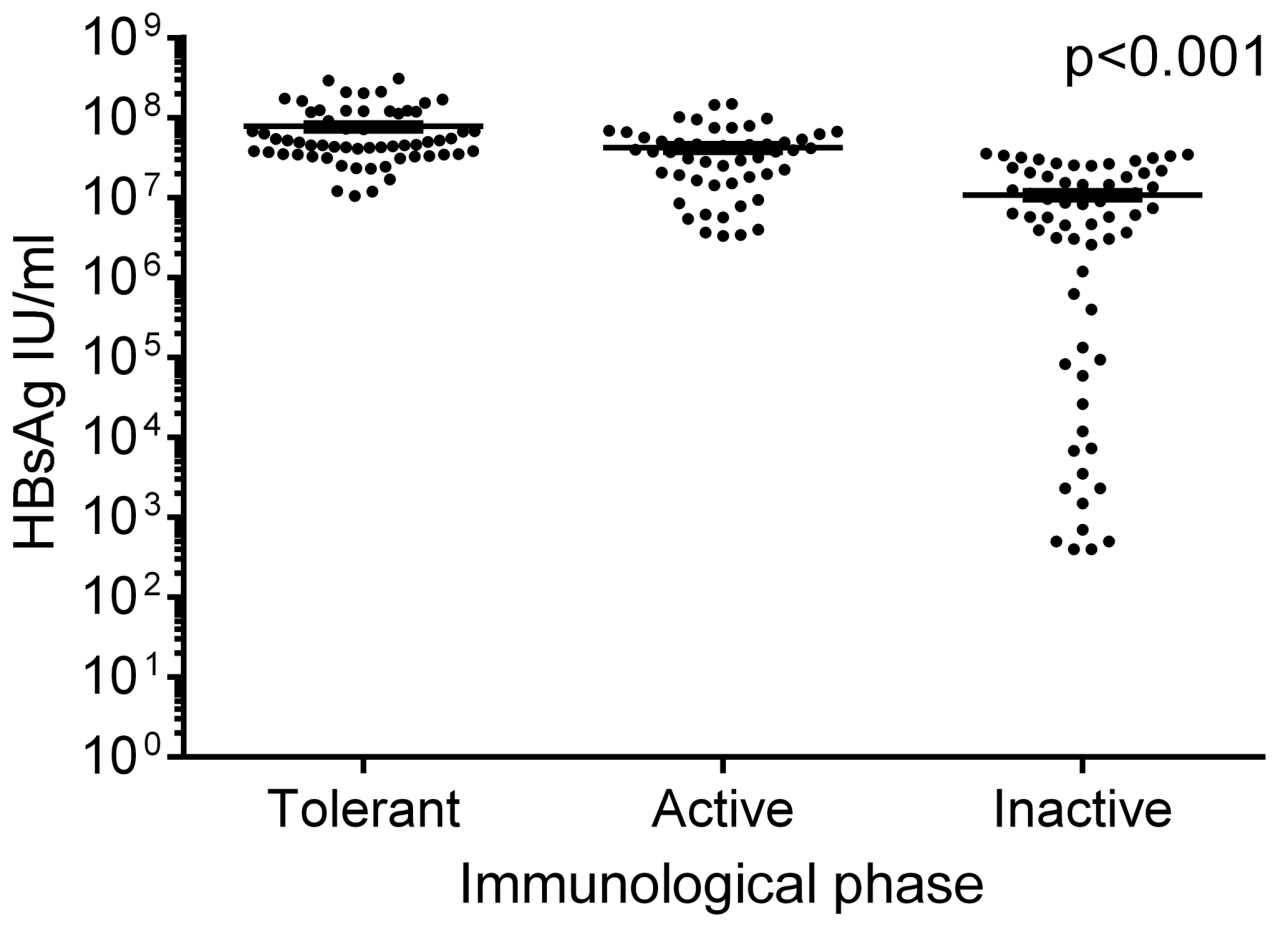
HBsAg quantity in different immunological phases of CHB. Plasma levels of HBsAg in children classified in different immunological phases of CHB were analysed. HBsAg levels were highest in plasma from immune-tolerant children, lower in plasma from immune-active children, and lowest in plasma from immune-inactive children, p<0.001. The range of HBsAg quantites was very broad in plasma from immune-inactive children. All the newly-seroconverted children showed plasma HBsAg levels in the upper range.

HBsAg was quantified in all plasma samples. First, we analysed HBsAg quantity in children classified in different phases of CHB. HBsAg levels were highest in plasma from immune-tolerant children, lower in plasma from immune-active children, and lowest in plasma from immune-inactive children, p<0.001. The correlation persisted when corrected for age, gender, and race, p<0.001.The range of HBsAg quantity was very broad in plasma from immune-inactive children. Interestingly, newly-seroconverted children all showed plasma HBsAg levels in the upper range. Figure 4. 

We also tested the association between plasma HBsAg levels and age and identified a significant correlation. However, when the effect of immunological phase on HBsAg plasma levels was considered, the significance did not persist. 

No association was found between HBsAg plasma quantity and gender or race. 

**Figure 5:**
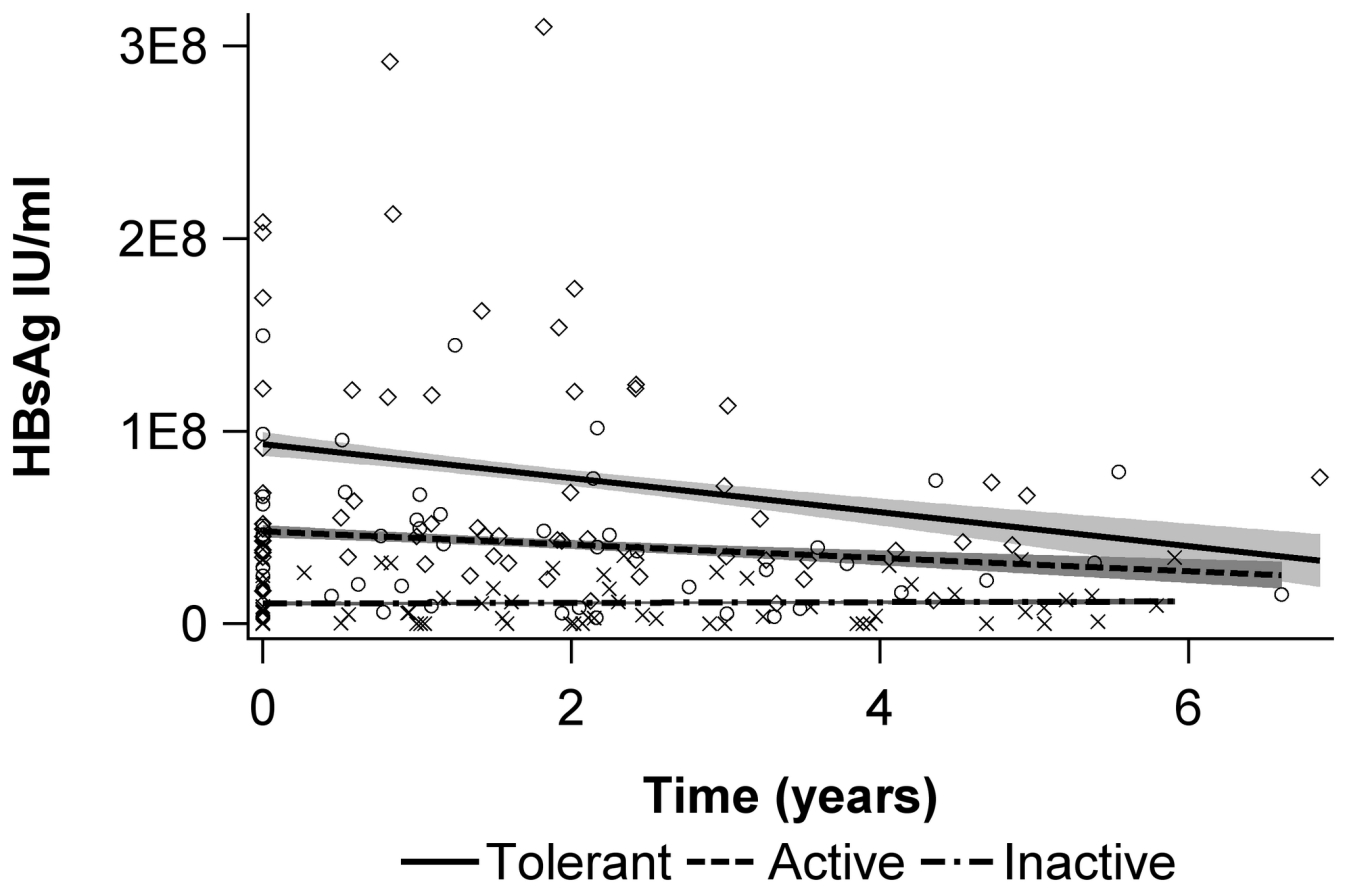
Quantitative changes in HBsAg over time in children with CHB. The quantitative changes in HBsAg over time in children with CHB were investigated. Within the individual child, plasma HBsAg levels decreased during the study period, p<0.001. The highest decrease was observed in immune-tolerant children, a lower decrease was observed in immune-active children, and HBsAg levels were stable in immune-inactive children, p<0.001. Bar: mean HBsAg; marked area: 95% confidence interval; time (years): the period from collection of the first sample from a given child.

Next, we investigated the quantitative changes in HBsAg over time. Within the individual child, plasma HBsAg levels decreased during the study period, p<0.001. The highest decrease was observed in immune-tolerant children, a lower decrease was observed in immune-active children, and HBsAg levels were stable in immune-inactive children, p<0.001. The results were adjusted for age. Figure 5.

### MicroRNAs, HBsAg, and HBV DNA

**Figure 6:**
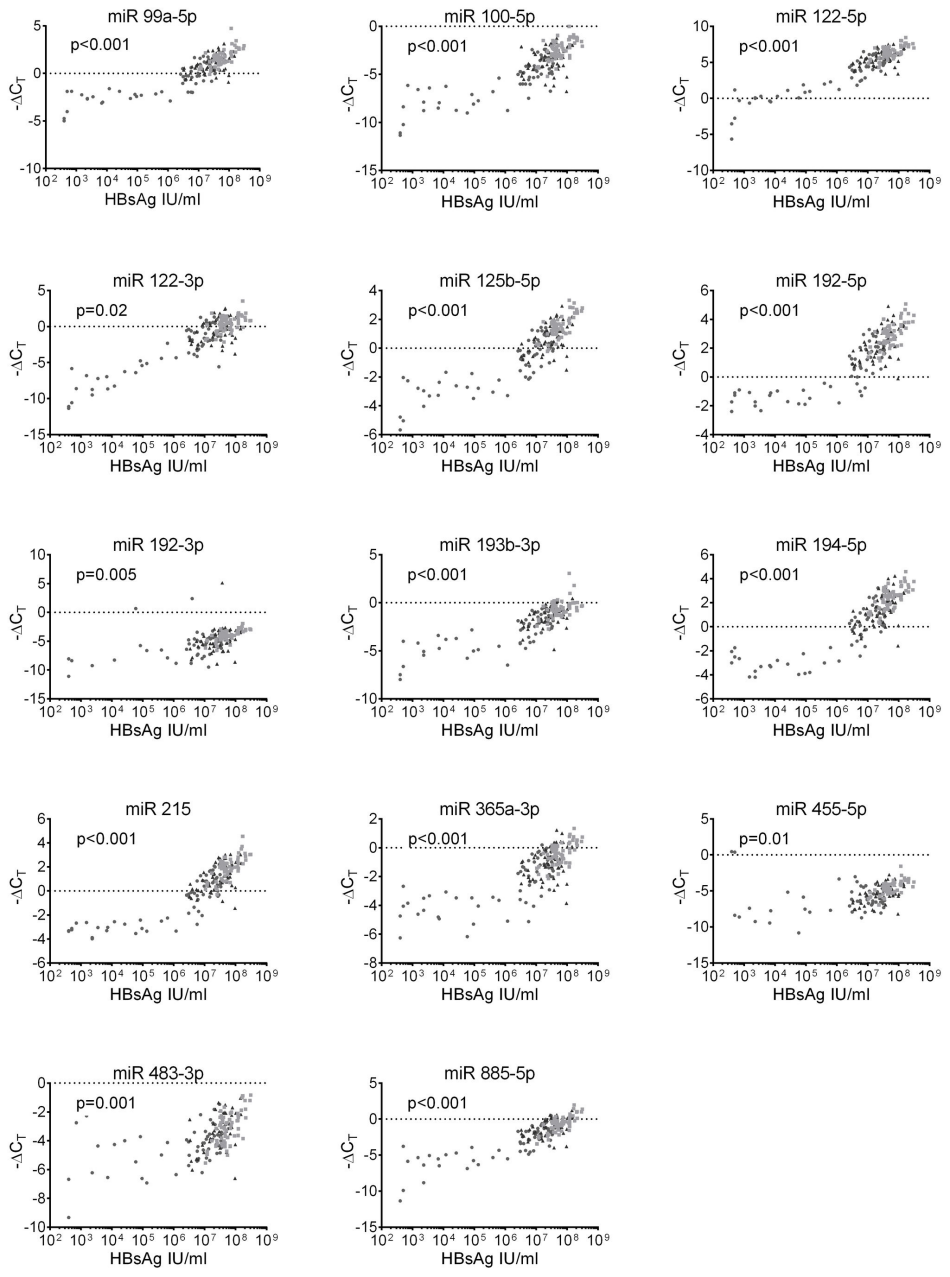
Correlation between circulating microRNAs and HBsAg. The quantitative relationship between circulating microRNAs and HBsAg was analysed. Highly-significant correlations were identified between the plasma levels of 11/14 microRNAs and HBsAg, p<0.004 (No significant association was observed between miR-122-3p, -192-3p, and –455-5p and HBsAg). Due to multiple testing only p<0.004 was regarded as significant.

The quantitative relationships between circulating microRNAs, HBsAg, and HBV DNA were analysed:

Highly-significant correlations (p<0.004) were identified between the plasma quantity of 11 out of 14 microRNAs and HBsAg (No significant association was observed between miR-122-3p, -192-3p, and –455-5p and HBsAg). Figure 6. 

We also found significant correlations between the plasma levels of all the microRNAs and HBV DNA (p<0.001) as well as between the quantity of HBsAg and HBV DNA (p<0.001). 

Multivariate analyses revealed the strongest correlation between microRNAs and HBsAg, p<0.001. Only miR-192-3p showed a stronger correlation with HBV DNA than with HBsAg. 

## Discussion

We followed a cohort of children with CHB over time with the aim of characterising selected plasma microRNAs and their correlation to HBsAg in the natural course of the disease.

Here, we show that the microRNAs investigated are actually significantly differentially expressed in different immunological phases of the disease in children. The microRNA plasma levels are highest in immune-tolerant children, lower in immune-active children, and reach the lowest values in immune-inactive children. These highly significant relationships between levels of circulating microRNAs and the immunological phases of CHB suggest a role of the identified microRNAs in the pathogenesis of CHB in children. Noteworthy, the biological functions of the microRNAs investigated are largely unknown, only miR-122 has been shown to inhibit HBV replication [10,28].

The present study is the first to characterise microRNA plasma levels in the natural history of CHB in children. We show that the plasma levels of four microRNAs (miR-99a-5p, -122-5p, -122-3p, and -125b-5p) decrease significantly over time in immune-tolerant and immune-active children whereas the microRNA plasma levels are stable in immune-inactive children. 

Chronic HBV infection is a dynamic condition, and liver damage is a result of complex interactions between virus and host. Our data might suggest that immunological control of the infection is achieved successively over time and that microRNA plasma levels are inversely correlated with immunological control of the disease. This hypothesis implies that the four microRNAs (miR-99a-5p, -122-5p, -122-3p, and -125b-5p) are better markers of immunological status than the remaining microRNAs investigated.

Assessing the severity of liver damage in children with CHB is a clinical challenge. ALT is a widely used biomarker of liver function, however, the sensitivity of ALT to diagnose virus-induced liver damage has been questioned [7,29]. Several studies identified miR-122 as a sensitive marker of liver damage, and recently a study using mouse models showed that plasma levels of miR-122 is a more sensitive biomarker for liver damage than ALT [30]. 

The present study reveal a highly significant correlation between plasma levels of miR-122 and the immunological phases of CHB, and no correlation is identified between levels of the circulating miR-122 and ALT. Only on excluding immune-tolerant children from analysis, a border significant correlation is observed between ALT and miR-122 plasma levels.

The suggestion that miR-122 is a more sensitive marker for minor liver damage than ALT would explain our surprising finding. A limitation of the present study is that only ALT was used to assess liver damage (liver biopsies are not indicated in asymptomatic children). Further studies are urgently needed to elucidate the sensitivity of miR-122 as a marker of minor liver damage in children with CHB as it may have major clinical implications. 

The mechanisms by which microRNAs are transferred from the cells to the bloodstream and the potential functions of plasma microRNAs in general are debated. Several studies suggest that circulating microRNAs are by-products of dead or dying cells and do not have any biological functions [31]. By contrast, mammalian cells in culture are shown to secrete microRNAs actively into the extra-cellular environment. Furthermore, it has been demonstrated that microRNAs carried by exosomes are biologically active once exosomes are internalised within cells. Plasma microRNAs might be involved in cell-cell communication [32-34]. We suggest that increased plasma microRNA levels in children with CHB are related to host-immune response and subsequent liver pathology. The present study however does not allow any conclusions on the biological functions of the microRNAs investigated. Nor conclusions on the specificity of the microRNAs can be drawn since no children with liver diseases other than CHB were included in the study.

It has been suggested that HBsAg is central in the complex interaction between virus and host [11,12]. We show for the first time that plasma HBsAg quantity varies significantly during different phases of CHB in children. The levels of HBsAg are highest in immune-tolerant children, lower in immune-active children, and lowest in immune-inactive children. These findings are in agreement with previous studies of HBsAg quantity in adults with CHB [35,36].

In the present study we furthermore reveal a significant decrease in HBsAg levels over time; the highest decrease is in immune-tolerant children, a lower decline in immune-active children, and HBsAg levels is stable in immune-inactive children. Natural history studies on adults with CHB have demonstrated that HBsAg levels decline slowly and progressively after HBeAg seroconversion [35,36]. Interestingly, we observe a broad range of HBsAg quantities in immune-inactive children and all the newly-seroconverted children show HBsAg levels in the upper range. The relatively short follow-up period of the present study may account for the discrepancy between our data and results of previous studies.

Subviral HBsAg particles are produced in large excess over infectious virions [13]. The use of this apparent excess production is yet to be understood. A recent study demonstrated that circulating HBsAg particles carry hepatocellular-derived microRNAs [14]. Interestingly, we reveal a highly-significant association between plasma levels of the majority of microRNAs investigated and HBsAg. A conceivable mechanism linking microRNAs and HBsAg might be that infected hepatocytes produce high amounts of microRNAs and HBV sequesters and expel these microRNAs inside HBsAg particles in order to evade host-immune response or, that HBV somehow triggers the release of microRNAs in order to attain optimal conditions for virus replication and HBsAg functions as a transporter of these microRNAs between cells. Further studies are needed to address these intriguing questions. 

To conclude, our data suggest that plasma levels of selected microRNAs and HBsAg are inversely correlated with immunological control of CHB in children. Four microRNAs may be better markers of immunological status than the remaining microRNAs investigated. 

Further studies are needed, however, to improve knowledge about the complex interaction between HBV and host, hopefully leading to the identification of future therapeutic targets and to enhanced management of children with CHB. 

## Supporting Information

Table S1 **Impact of extended processing time from sample collection to plasma isolation on plasma microRNA levels.**
(DOC)
