# Supplementary material for: Hepatitis B Surface Antigen Quantity Positively Correlates with Plasma Levels of microRNAs Differentially Expressed in Immunological Phases of Chronic Hepatitis B in Children
Source: PLoS One. 2013 Nov 11;8(11):e80384. doi: 10.1371/journal.pone.0080384 (PMC3823657; doi:10.1371/journal.pone.0080384)
Supplement: Table S1 — Impact of extended processing time from sample collection to plasma isolation on plasma microRNA levels. (DOC) [file pone.0080384.s001.doc]

Table S1, Impact of extended processing time from sample collection to plasma isolation on plasma microRNA levels.

| **microRNA** | **Immunological phase of CHB** | **Mean –ΔC_T_ (±SD)** | | **P-value** |
| --- | --- | --- | --- | --- |
|  |  | **<4 hours** | **>4 hours** |  |
| **99a-5p** | Tolerant | 1.8 (0.6) | 1.6 (1.2) | 0.09 |
|  | Active | 1.1 (0.95) | 0.76 (1.2) | 0.21 |
|  | Inactive | -0.85 (1.3) | -0.51 (1.9) | 0.16 |
| **100-5p** | Tolerant | -2.2 (0.69) | -2.8 (1.4) | 0.086 |
|  | Active | -2.8 (0.98) | -3.8 (1.6) | 0.13 |
|  | Inactive | -5.7 (1.8) | -5.3 (2.5) | 0.17 |
| **122-5p** | Tolerant | 6.6 (0.58) | 6.3 (1.2) | 0.39 |
|  | Active | 5.8 (1.05) | 5.3 (1.3) | 0.074 |
|  | Inactive | 3.2 (2.1) | 3.3 (3.1) | 0.26 |
| **122-3p** | Tolerant | 0.56 (0.88) | 0.31 (1.4) | 0.41 |
|  | Active | 0.12 (1.4) | -0.9 (2.0) | 0.07 |
|  | Inactive | -3.2 (2.4) | -2.8 (4.0) | 0.097 |
| **125b-5p** | Tolerant | 1.6 (0.58) | 1.3 (1.1) | 0.17 |
|  | Active | 1.0 (0.95) | 0.33 (1.3) | 0.08 |
|  | Inactive | -1.2 (1.4) | 0.82 (2.0) | 0.13 |
| **192-5p** | Tolerant | 3.1 (0.68) | 2.8 (1.1) | 0.23 |
|  | Active | 2.5 (0.99) | 2.0 (1.3) | 0.09 |
|  | Inactive | 0.25 (1.4) | 0.8 (1.8) | 0.15 |
| **192-3p** | Tolerant | -3.8 (0.64) | -4.1 (1.2) | 0.051 |
|  | Active | -4.6 (1.4) | -4.9 (3.1) | 0.26 |
|  | Inactive | -5.8 (1.9) | -5.7 (2.5) | 0.25 |
| **193b-3p** | Tolerant | -0.61 (0.51) | -0.64 (1.4) | 0.45 |
|  | Active | -0.71 (0.84) | -1.6 (1.4) | 0.12 |
|  | Inactive | -3.2 (1.5) | -2.7 (2.0) | 0.11 |
| **194-5p** | Tolerant | 2.6 (0.67) | 2.1 (1.2) | 0.09 |
|  | Active | 2.0 (0.98) | 0.92 (1.7) | 0.07 |
|  | Inactive | -1.0 (1.9) | -0.3 (2.1) | 0.068 |
| **215** | Tolerant | 2.1 (0.68) | 1.7 (1.2) | 0.054 |
|  | Active | 1.8 (0.98) | 0.85 (1.5) | 0.053 |
|  | Inactive | -1.1 (1.6) | -0.45 (2.0) | 0.11 |
| **365a-3p** | Tolerant | -0.26 (0.6) | -0.59 (0.92) | 0.11 |
|  | Active | -0.93 (1.03) | -1.5 (1.2) | 0.061 |
|  | Inactive | -3.0 (1.4) | -2.1 (1.9) | 0.059 |
| **455-5p** | Tolerant | -4.4 (0.65) | -4.8 (1.3) | 0.09 |
|  | Active | -5.0 (1.1) | -5.8 (1.5) | 0.071 |
|  | Inactive | -6.6 (1.5) | -5.7 (2.3) | 0.11 |
| **483-3p** | Tolerant | -2.8 (1.2) | -3.6 (1.6) | 0.071 |
|  | Active | -3.0 (1.5) | -3.9 (1.4) | 0.09 |
|  | Inactive | -4.4 (1.2) | -4.4 (1.7) | 0.91 |
| **855-5p** | Tolerant | -0.44 (0.89) | -1.1 (1.3) | 0.071 |
|  | Active | -0.61 (1.07) | -2.0 (1.6) | 0.05 |
|  | Inactive | -3.5 (1.8) | -3.2 (2.8) | 0.1 |

Footnote: Samples from 23/42 children with CHB were processed for plasma isolation within 4 hours of collection whereas processing of samples from the remaining 19/42 children with CHB were delayed up to 48 hours due to shipping. Non-parametric Mann-Whitney test were used to determine statistical significance.
